# Identification of a novel *GJA3* mutation in a large Chinese family with congenital cataract using targeted exome sequencing

**DOI:** 10.1371/journal.pone.0184440

**Published:** 2017-09-06

**Authors:** Yihua Yao, Xuedong Zheng, Xianglian Ge, Yanghui Xiu, Liu Zhang, Weifang Fang, Junzhao Zhao, Feng Gu, Yihua Zhu

**Affiliations:** 1 The First Affiliated Hospital of Fujian Medical University, Fuzhou, Fujian, China; 2 School of Ophthalmology and Optometry, Eye Hospital, Wenzhou Medical University, State Key Laboratory Cultivation Base and Key Laboratory of Vision Science, Ministry of Health and Zhejiang Provincial Key Laboratory of Ophthalmology and Optometry, Wenzhou, Zhejiang, China; 3 The Second Affiliated Hospital and Yuying Children’s Hospital of Wenzhou Medical University, Wenzhou, Zhejiang, China; Tsinghua University School of Life Sciences, CHINA

## Abstract

Autosomal dominant congenital cataract (ADCC) is a clinically and genetically heterogeneous ocular disease in children that results in serious visual impairments or even blindness. Targeted exome sequencing (TES) is an efficient method used for genetic diagnoses of inherited diseases. In the present study, we used a custom-made TES panel to identify the genetic defect of a four-generation Chinese family with bilateral pulverulent nuclear cataracts. A novel heterozygous missense mutation c.443C>T (p. T148I) in *GJA3* was identified. The results of the bioinformatic analysis showed that the mutation was deleterious to the structure and hemichannel function of Cx46 encoded by *GJA3*. Plasmids expressing wild-type and mutant human Cx46 were constructed and ectopically expressed in human lens epithelial cells (HLECs) or human embryonic kidney (HEK-293) cells. Fluorescent images indicated aggregated signals of mutant protein in the cytoplasm, and a higher protein level was also detected in T148I stable cell lines. In summary, we identified a novel mutation in *GJA3* for ADCC, which provided molecular insights into the pathogenic mechanism of ADCC.

## Introduction

Congenital cataract (CC) is a common and severe hereditary ocular disease in children, leading to serious visual impairments or even blindness[1, 2]. The main characteristic of CC is lens opacity and abnormal ocular development from birth or during infancy, childhood or adolescence. Approximately 1 to 6 cases per 10,000 live births develop non-syndromic CC in industrialized countries, whereas these proportions are estimated to be 5 to 15 per 10,000 live births in developing countries[3–5]. Approximately 8% to 25% of isolated CC are considered to be hereditary diseases, accounting for nearly 70% of congenital cataracts[6]. Autosomal dominant congenital cataract (ADCC) is the most common trait of CC patients, and the disease can also be inherited through autosomal recessive or X-linked models[7].

To date, more than 60 genetic loci for inherited CCs have been mapped. Among these, more than 20 causative genes for these loci have been associated with ADCC, and the number of the identified genes is increasing[7, 8]. Half of the identified mutations in the ADCC family are crystallin genes, including *CRYAA*, *CRYAB*, *CRYBA1/A3*, *CRYBA4*, *CRYBB1*, *CRYBB2*, *CRYBB3*, *CRYGC*, *CRYGD* and *CRYGS*, another one-quarter are gap junction (GJ) genes, including *GJA3* and *GJA8*[9]. The remaining genes are *MIP*, *LIM2*, *MP19*, *BFSP1/2*, *HSF4*, *MAF*, *PITX3* and *EPHA2*[7, 9–11].

GJs are transport channels that function by forming extensive intercellular communication channels in the avascular lens; each channel is created by two hemichannels, also known as connexons, between adjacent cells[12]. GJs provide an intracellular and extracellular transmembrane network for the transportation of small molecules, such as second messengers, nutrients and ions. The functions of GJs are not only crucial to the maintenance of lens homeostasis and transparency but also important for lens development and differentiation[13]. Cx43, Cx46 and Cx50, encoded by *GJA1*, *GJA3* and *GJA8*, respectively, are mainly located in lens fiber cells as well as partly in HLECs[14, 15]. Mutations of *GJA3* and *GJA8* are linked to ADCC through different mechanisms, including the formation of inefficient GJ channels[16–18], abnormal expression in the cytoplasm or nucleus[19, 20], changes in channel or hemichannel functions[21], alterations in electrophysiological characteristics[22], and dominant negative mutants on wild-type (WT) GJs[23, 24]. Therefore, mutated GJ genes (including *GJA3* identified in this study) are the main disease-causing genes in CC patients.

In this study, the genetic defect of a four-generation Chinese family with pulverulent nuclear ADCC were identified via TES and Sanger sequencing. Using this approach, a novel heterozygous mutation in *GJA3* was revealed in this family. Silico prediction and experimental studies were performed to dissect the pathogenic mechanism of this mutation associated with ADCC.

## Materials and methods

### Ethics statement

This study was approved by the ethics committee of the First Affiliated Hospital of Fujian Medical University, Fuzhou, China, on March 9, 2016. Participants were recruited from April to June 2016 and provided written informed consent consistent with the tenets of the Declaration of Helsinki.

### Patients and preparation of genomic DNA

A four-generation Chinese family with ADCC was recruited for clinical evaluations, and the extraction of genomic DNA was completed in the First Affiliated Hospital of Fujian Medical University of China. Thirty-four participants took part in this study, including 5 affected and 29 unaffected individuals. All the family members underwent detailed physical and ophthalmologic examinations. Genomic DNA used for polymerase chain reaction (PCR) amplification and TES analysis was extracted from peripheral blood leukocytes following the instructions of a DNeasy Blood and Tissue Kit (QIAGEN; USA) and quantified with a NanoDrop instrument (Thermo Fisher Scientific, USA).

### Target exome capture sequencing and bioinformatic analysis

A total of 134 genes related to CC and other ophthalmic diseases were investigated (S1 Table) based on the Online Mendelian Inheritance in Man (https://www.ncbi.nlm.nih.gov/omim) and published studies. The coding exons and flanking regions of these genes were captured and enriched for the library using a custom-made target exome capture panel (MyGenostics, Beijing, China). Briefly, a minimum of 2 μg of proband DNA was fragmented into 50–100 base pairs (bp) and prepared for the Illumina library according to the manufacturer’s recommendations. Following quality control of the enriched fragments, high-throughput sequencing was performed on an Illumina NextSeq500 system (Illumina, USA). The clean reads were aligned in reference to the UCSC human reference genome (GRCh37/hg19, http://genome.ucsc.edu) using the Burrows Wheeler Aligner program (http://bio-bwa.sourceforge.net/bwa.shtml). Variants, including SNPs, indels and block substitutions, were analyzed by GATK and annotated using the 1000 Genomes Project, ESP6500, ExAC, the HGMD database and the MyGenostics local database. The probable pathogenic mutations were collected and evaluated using the following online software: Sorting Intolerant from Tolerant (SIFT, http://sift.jcvi.org/), Polymorphism Phenotyping (PolyPhen-2, http://genetics.bwh.harvard.edu/pph2/), and Mutation Taster (http://mutationtaster.org/). Homologous model structures of WT and mutant *GJA3* were predicted with the Swiss-Model program (https://www.swissmodel.expasy.org/) and visualized using RasMol software (version 2.7.5.2) based on the template of the resolved structure of connexin-26 (http://www.rcsb.org/pdb, No. 2ZW3). Hydropathic characters for amino acid changes were displayed with Kyte-Doolittle Hydropathy Plot online software (http://gcat.davidson.edu/DGPB/kd/kyte-doolittle.htm).

### Validation of candidate mutations and segregation analysis

Specific primer pairs were designed to amplify regions harboring the point mutations via the IDT PrimerQuest Tool (http://sg.idtdna.com/Primerquest). PCR and Sanger sequencing were applied to validate the candidate *GJA3* mutation in all participants and controls (forward-primer: 5’- CGCCCACCCTCATCTACCT-3’; reverse-primer: 5’- GTGGGAACCCGATGGCAAC-3’). Purified PCR products were sequenced on an ABI3730 Automated Sequencer (PE Biosystems, USA). Sequencing data were compared with the reference sequences in the NCBI gene bank using Chromas software as well as reported mutations in the literature.

### Plasmid construction

The coding sequence from exon 2 of the *GJA3* gene was amplified from genomic DNA by PCR. WT *GJA3* coding fragments were prepared using the Fast HiFidelity PCR kit (TIANGEN, Beijing, China) and the following primers: forward primer (5’-TGCCGGAATTCTGATGGGCGACTGGAGCTTTC-3’) and reverse primer (5’-TAGTAGGATCCCGGATGGCCAAGTCCTCCG-3’). After digestion with EcoRI and BamHI (NEB, USA), the purified PCR products were inserted into the eukaryotic expression vector pEGFP-N1. A C-terminus Flag-tag was added to the coding region of *GJA3* and inserted into another plasmid (pSin) as previously described[19, 25]. The Cx46T148I and Cx46G143R mutants were generated using the QuikChangeTM Site-Directed Mutagenesis Kit (Stratagene, USA) with the following primers: Cx46T148I forward primer (5’-GGGCGCTGCTGCGGATTACGTCTTCAACATC-3’) and Cx46T148I reverse primer (5’-GATGTTGAAGACGTAATCCGCAGCAGCGCCC-3’), Cx46G143R forward primer (5’-AGGGTGCGCATGGCCAGGGCGCTGCTGCGGA-3’) and Cx46G143R reverse primer (5’-TCCGCAGCAGCGCCCTGGCCATGCGCACCCT-3’). Vectors of pSin-Cx46WT, pSin-Cx46T148I, pEGFP-N1-Cx46WT, pEGFP-N1-Cx46-T148I and pEGFP-N1-Cx46G143R were confirmed by Sanger sequencing before transfection.

### Cell culture and transfection

HLECs and HEK-293 cells were cultured in Dulbecco’s Modified Eagle’s Medium supplemented with 10% fetal bovine serum and 100 mg/ml of penicillin and 100 mg/ml of streptomycin (Gibco, USA) in a 37°C incubator with 5% CO_2_. The pEGFP-N1-Cx46WT, pEGFP-N1-Cx46-T148I and pEGFP-N1-Cx46G143R were transiently expressed in HLECs for the analysis of Cx46 cellular localization. HEK-293 was seeded at day 0 and transfected with pSin-*GJA3*/Flag WT or mutant vectors after 24 hours using TurboFect Transfection Reagent (Thermo Fisher Scientific, Lithuania) according to the manufacturer’s instructions. HEK-293 cell lines with stably expressed WT or mutant *GJA3*/Flag were obtained after these puromycin-resistant cells were screened with 1 μg/ml puromycin (Sigma, USA) for two weeks. Cell growth curves were assessed via the Cell Counting Kit-8 kit (Beyotime, China) according to the manufacturer’s protocol.

### Fluorescence microscopy analysis

HLECs were cultured on cell slides in six-well dishes until reaching 60% confluence 24 hours before transfection. For the cellular localization of *GJA3*/EGFP fusion protein, cells were washed three times with PBS 48 hours after transfection and then fixed with 4% paraformaldehyde for 15 min, permeabilized with 0.1% Triton X-100 for 15 min, and subjected to nuclei labeling with DAPI (Beyotime, China) for 15 min. Cells were washed three times between each step. Finally, fluorescent images were captured in the same exposure using DMi8 Microsystems in FITC and DAPI channels (Leica, Germany). The percentages of cells with aggregation were calculated from ten random fluorescence fields in a single-blinded method as previously described[26]. The percentages of the Cx46WT, Cx46T148I and Cx46G143R cells with aggregations and the total EGFP-positive cells were calculated by Image J software.

To quantify plaques formation, the percentages of cell pairs with GJ plaques to all cell pairs expressing *GJA3*/EGFP fusion protein was calculated as previously described[27]. The results are given as average percentages of cell pairs with GJ plaques.

For dye uptake experiments, three groups of HEK-293 cells (control, Cx46WT and Cx46T148I) were seeded in 12-well plates for hemichannel function analysis. Until reaching over 60% confluency, the cells were washed twice with a D-Hanks (Solarbio, China) solution that did not contain Ca^2+^ or Mg^2+^ and then incubated in D-Hanks or D-Hanks containing 1.2 mM Ca^2+^ where hemichannels were close[28] or a GJ blocker, flufenamic acid (FFA, 300 mM, Sigma, USA)[28, 29]. The solutions utilized for incubation contained 0.1% DAPI and were removed after 10 min. The cells were washed two times with preheated PBS. Immunocytochemical staining of Cx46/Flag procedures were performed according to the manufacturer’s protocol with a 1:500 dilution of anti-Flag mouse monoclonal antibody (Sigma, CAS No. f1804, USA) and then staining with a 1:200 dilution of goat anti-mouse IgG-FITC (Santa Cruz, CAS No. sc-2010, USA). Cells loaded with dye were counted via DAPI channel, and Cx46/Flag positive cells were displayed in FITC channel. At least 30 fields were captured for each group, and dye uptake experiments were repeated three times. The percentages of DAPI-loaded cells were measured and quantified by Image J software. Fluorescence microscopy experiments were repeated at least three times. Student’s t-test was applied to identify statistical differences.

### Western blotting analysis

HLECs transiently transfected with plasmids or stable HEK-293 cells lines were harvested, and total protein was extracted using a RIPA protein extraction kit (Beyotime, China). Proteins were separated by 12% SDS-polyacrylamide gels and individually transferred to methanol-activated polyvinylidene difluoride membranes (Millipore, USA). The membranes were immunoblotted with a 1:1000 dilution of anti-Flag mouse monoclonal antibody (Sigma, CAS No. f1804, USA) or a 1:200 dilution of anti-Cx43 rabbit polyclonal antibody (Santa Cruz, CAS No. sc-9059, USA). A 1:1000 dilution of anti-GAPDH rabbit antibody (Diagbio, CAS No.db106, China) was used as an internal control. The fluorescent secondary goat anti-mouse (LI-COR Biosciences, CAS No.926-68072, USA) or goat anti-rabbit (LI-COR Biosciences, CAS No.925-32211, USA) antibodies (1:5000) were used for visualization. The signals were analyzed using the Image J program. The target protein expression levels were normalized relative to GAPDH expression. Each experiment was repeated at least three times.

## Results

### Clinical evaluation

**Fig 1:**
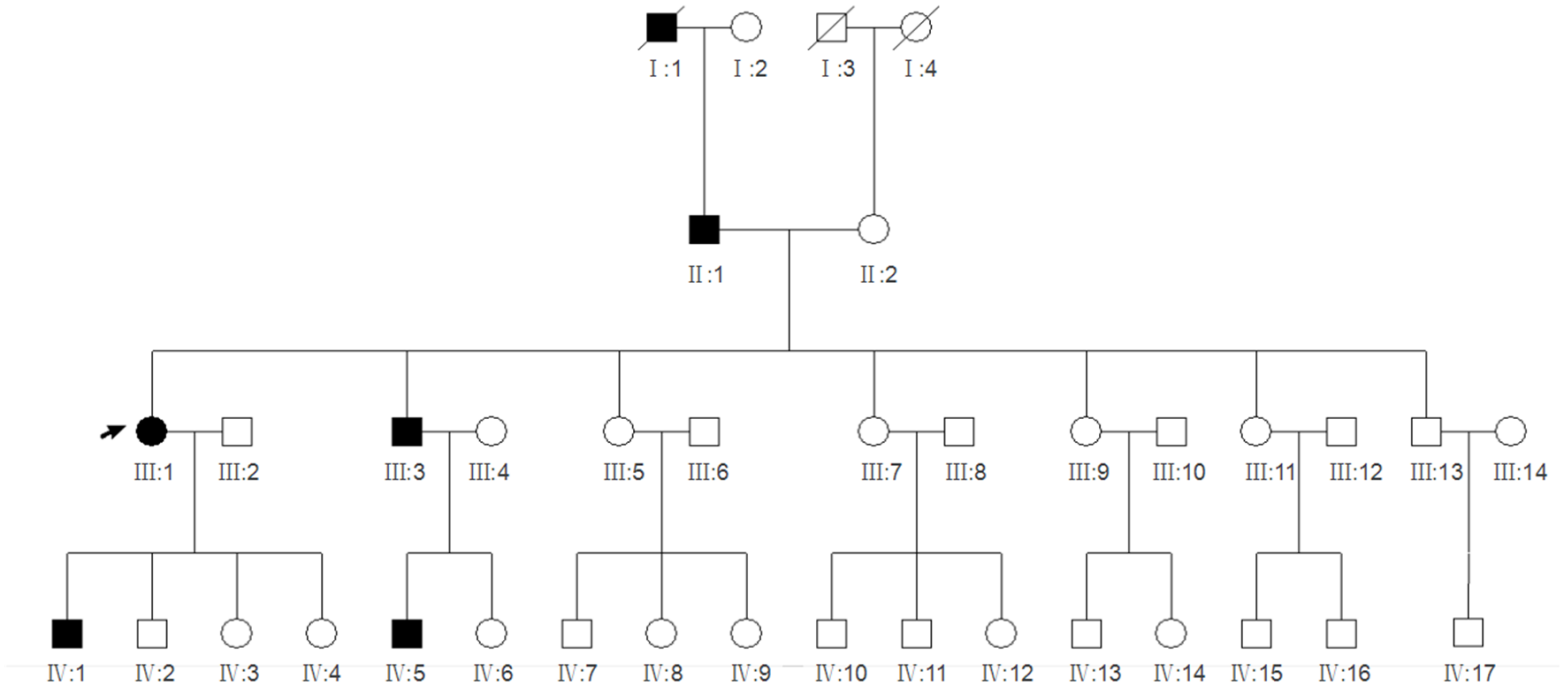
Pedigree of this ADCC family. Cataract pedigree. Squares and circles symbolize males and females, respectively. Black and white lines denote affected status and unaffected status, respectively. A solid black arrow indicates the proband (III:1), and the diagonal lines indicate deceased family members.

**Fig 2:**
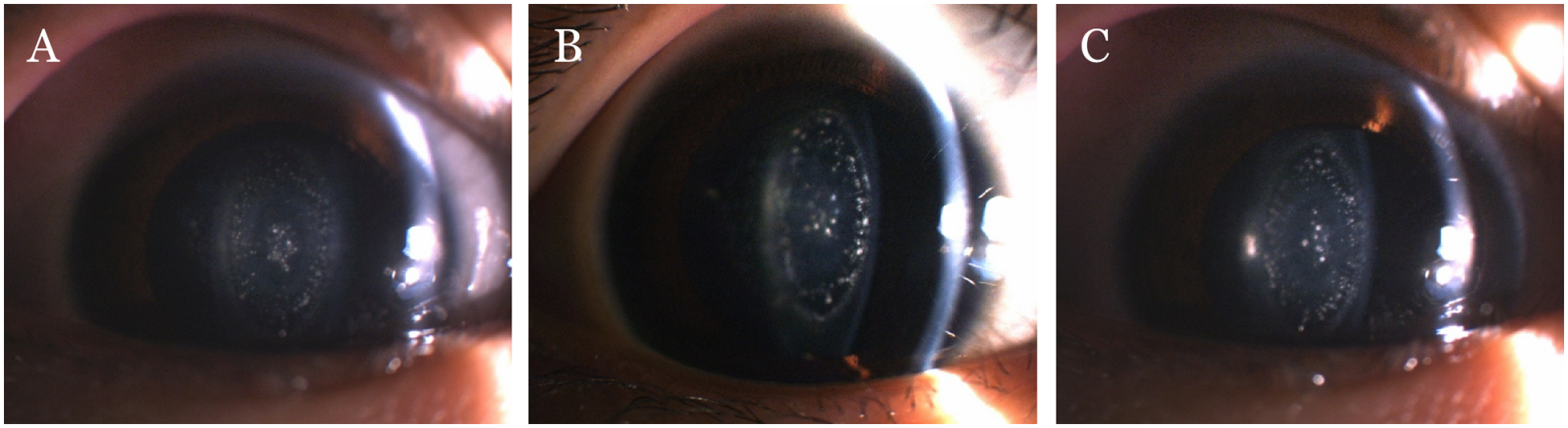
Photographs of affected individuals in this family. (A) the proband (III:1), (B) her older brother (III:3) and (C) the proband’s son (IV:5) showed bilateral pulverulent nuclear cataracts.

A Chinese family with four generations of individuals (five affected individuals and twenty-nine unaffected members) was enrolled in this study and diagnosed with ADCC (Fig 1). The proband (III:1) was a 35-year-old female who was diagnosed with bilateral pulverulent nuclear cataracts and had undergone cataract phacoemulsification and intraocular lens implantation in both eyes at approximately 30 years of age. The ages of the other four affected members (II1, III:3, IV:1, IV:5) ranged from 9 to 55 years, and they had varying lens opacities due to bilateral pulverulent nuclear cataracts. All patients complained of gradual blurred vision starting at approximately age 10, and the phenotype of ADCC was bilateral pulverulent nuclear opacities (Fig 2). No other ocular or systemic abnormalities were present in this pedigree.

### Candidate mutations of targeted exome sequencing (TES) and validation analysis

**Fig 3:**
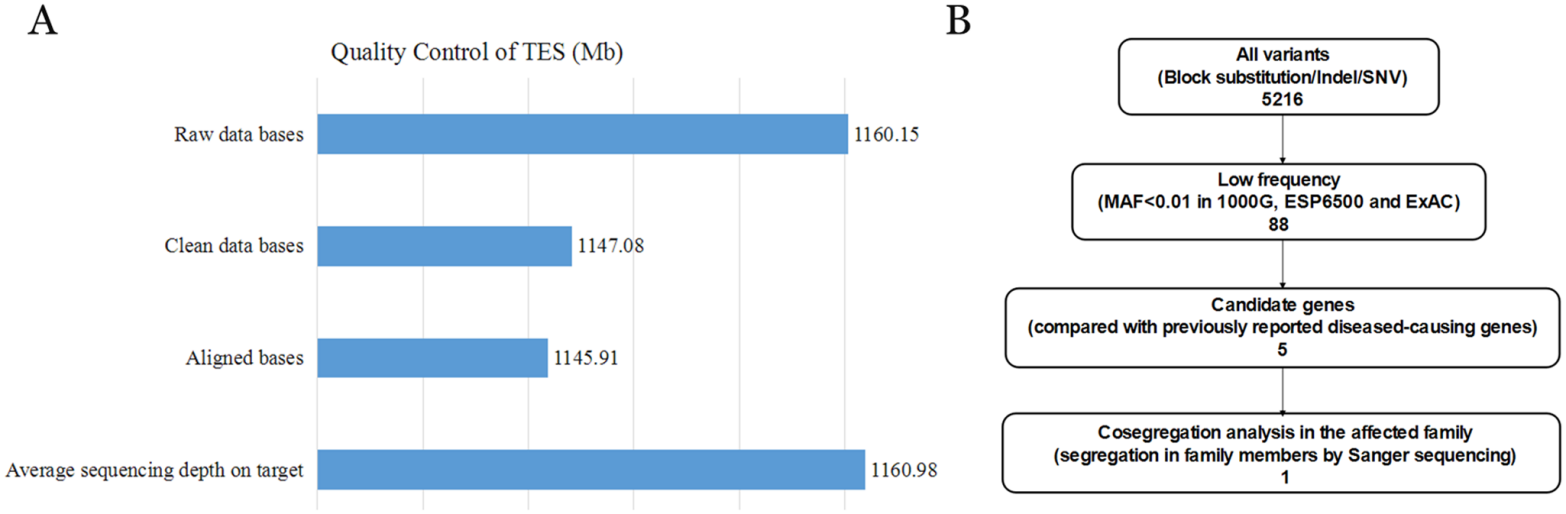
TES filter process and quality control in this study. (A) The high-quality data from TES in the proband were sufficient for the mutation screen and validation. A total of 1147.08 Mb of clean data were obtained after NGS. The coverage of all targeted regions was 99.86%, while designed targeted exomes in the 20× read were 98.56%; those in the 10× read were 99.46%; and those in the 4× read were 99.76%. (B) Schematic representation of the filter strategies employed in our study. A heterozygous mutation in *GJA3* was detected via TES based on database and bioinformatic analyses.

**Fig 4:**
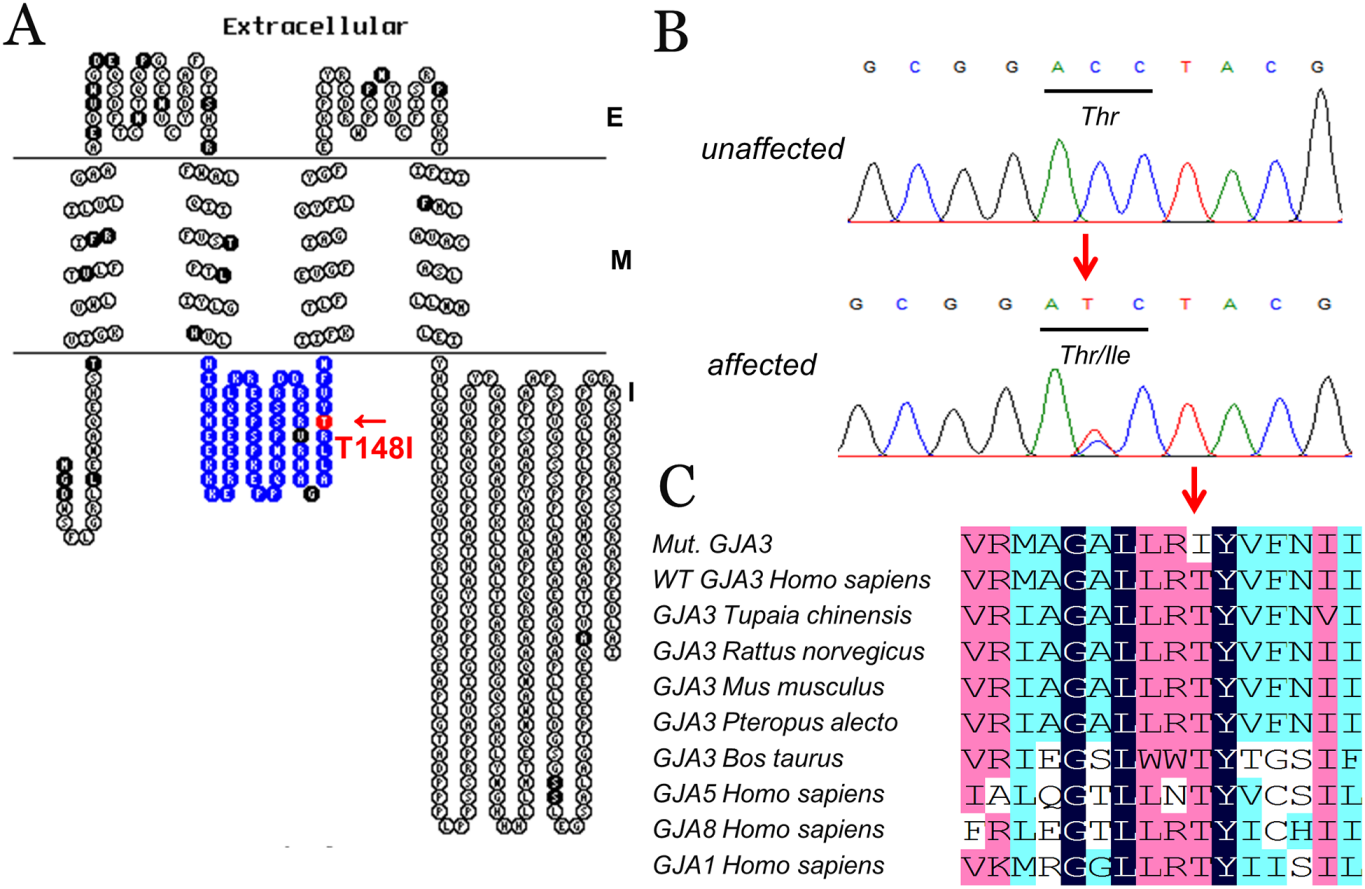
T148 is a well-conserved amino acid of Cx46 and *GJA3*/p. T148I is a novel mutation with co-segregation in this family. (A) *GJA3*/p. T148I is located at the cytoplasmic loop (indicated by the blue square) domain of the Cx46 protein. The membrane topological structure of Cx46 was generated by TOPO2 software. This mutation (indicated by the red square) is located in the cytoplasmic loop domain; the black square indicates reported mutations associated with CCs (E: extracellular; M: membrane; I: intracellular). (B) Sanger sequencing results showed that this missense mutation was detected in all affected individuals and was not shown in unaffected family members or in the 100 unrelated control individuals. (C) Multiple protein sequence alignments. Multiple sequence alignments of *GJA3* from different species and *GJA* family members (including *GJA1*, *GJA5*, and *GJA8*) from a human revealed that codon 148, where the mutation (p. T148I) occurred, was located within a highly conserved region. The “mut.” sequence indicates the sequence with the mutation detected in this family.

A custom-made TES panel was used to detect the genetic defect in this ADCC family. A total of 1160.98 Mb of raw data of average sequencing depth on target and 1147.08 Mb of clean data bases were obtained after TES. Coverage of all targeted regions was 99.86%, designed targeted exomes in the 20× read were 98.56%, while those in the 10× read were 99.46% and those in the 4× read were 99.76%. These high-quality data indicated that TES in this proband was sufficient for the mutation screen (Fig 3A). A minor allele frequency (MAF) less than 0.01 was used for filtration of existing mutations in the 1000 Genomes Project, ESP6500, ExAC, HGMD and the MyGenostics local database. A total of 5216 variants were detected in the proband, including the block substitution, indels and SNPs. After filtering out the high-frequency variants in the database, eighty-eight low-frequency variants were obtained. Finally, five candidate variants and only one confirmed variant were identified when compared with previously reported disease-causing genes as well as co-segregation in these family members by Sanger sequencing (Fig 3B). Taken together, these databases of TES revealed a heterozygous *GJA3* mutation located in the second exon of this gene associated with the Chinese ADCC family. Aligning sequences with multiple species by multiple sequence alignment (DNAMAN, version 6.0.40) revealed that the threonine at the 148th amino acid of Cx46 is a highly conserved residue (Fig 4A and 4C). Sanger sequencing revealed that this missense mutation was detected in all affected individuals and was not shown in unaffected family members or in the 100 unrelated control individuals (Fig 4B).

### Bioinformatic analysis and 3D homologous model structures

**Fig 5:**
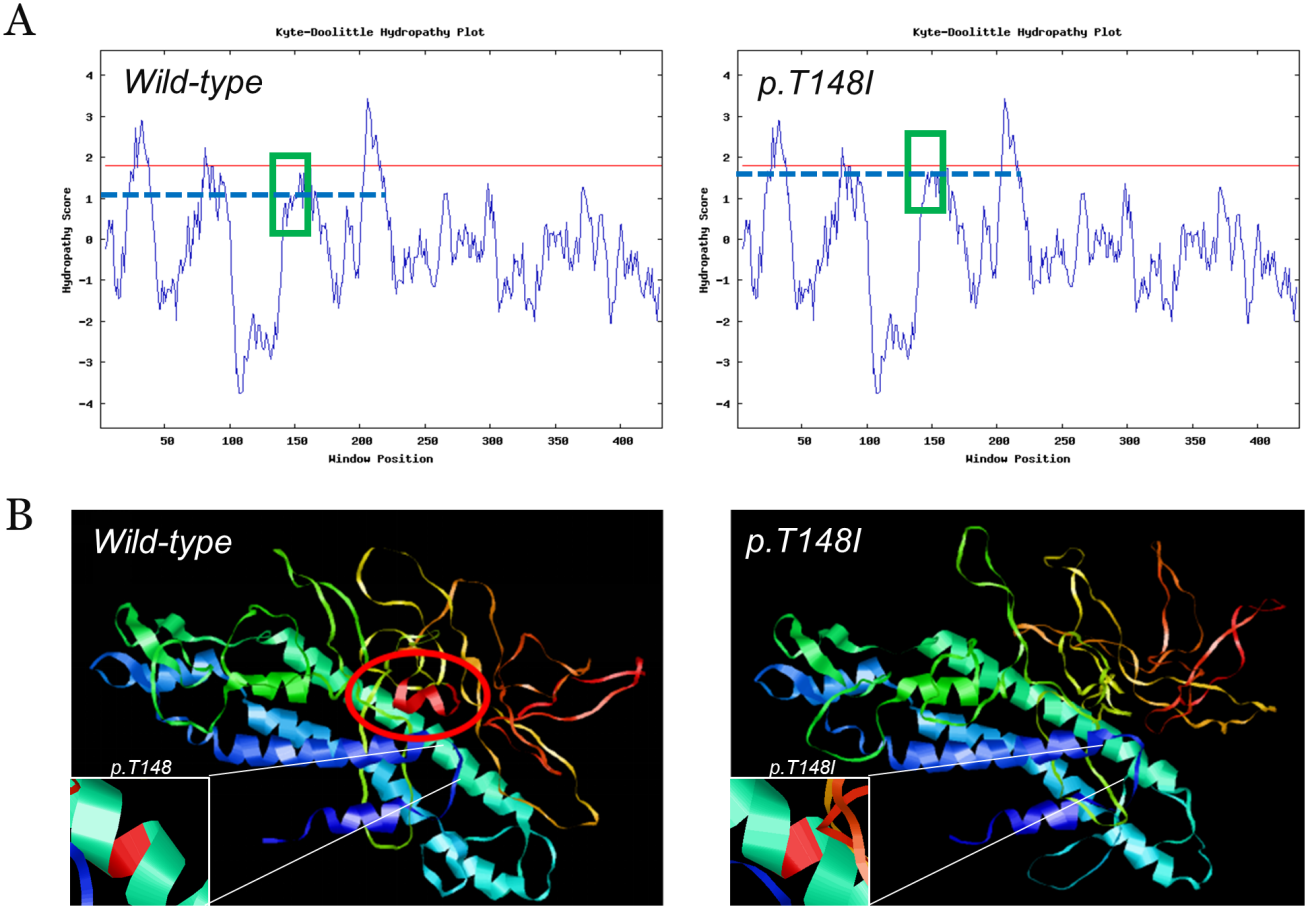
Hydrophilicity analysis of the WT and mutant proteins as well as homologous modeling predicted by the Swiss-Model program. (A) The hydropathic character of the changes in the mutant protein indicated a higher hydrophobicity than that of the WT. (B) The conformation of mutant Cx46 underwent a significant change with an α-helix deletion in the C-terminal when compared with that of the WT model. The α-helix deletion was marked in red circle and the locations of amino acid 148 were marked in red.

The effect of the missense mutation in p. T148I was predicted to be deleterious by SIFT with a score of -5.492 (a score ≤-2.5 is considered a deleterious variant) and probably damaging by PolyPhen-2 with a score of 0.989 (sensitivity: 0.72; specificity: 0.97). This was also predicted to be a disease-causing mutation by MutationTaster with a score of approximately 0.9999. The hydropathic character with regard to changes in the mutant protein indicated a higher hydrophobicity than that of the WT (Fig 5A). Homologous modeling predicted by the Swiss-Model program and RasMol software showed that the conformation of the mutant Cx46 underwent a great change with an α-helix deletion in the C-terminus when compared with that of the WT model (Fig 5B). Taken together, the bioinformatic analysis revealed that the p. T148I mutation was a novel disease-causing mutation.

### Functional analysis

Molecular alterations in the p. T148I mutant in this ADCC family were studied using mutant-specific EGFP and Flag-tag vectors. WT or mutant coding regions from human *GJA3* were cloned into the eukaryote expression vectors pEGFP-N1 and pSin, respectively. According to different transfection efficiencies in HLECs and HEK-293, vectors of pEGFP-N1-*GJA3* were used to detect the intracellular localization via fluorescence microscopy, while vectors of pSin-*GJA3* were used to detect the hemichannel function and protein expression level.

**Fig 6:**
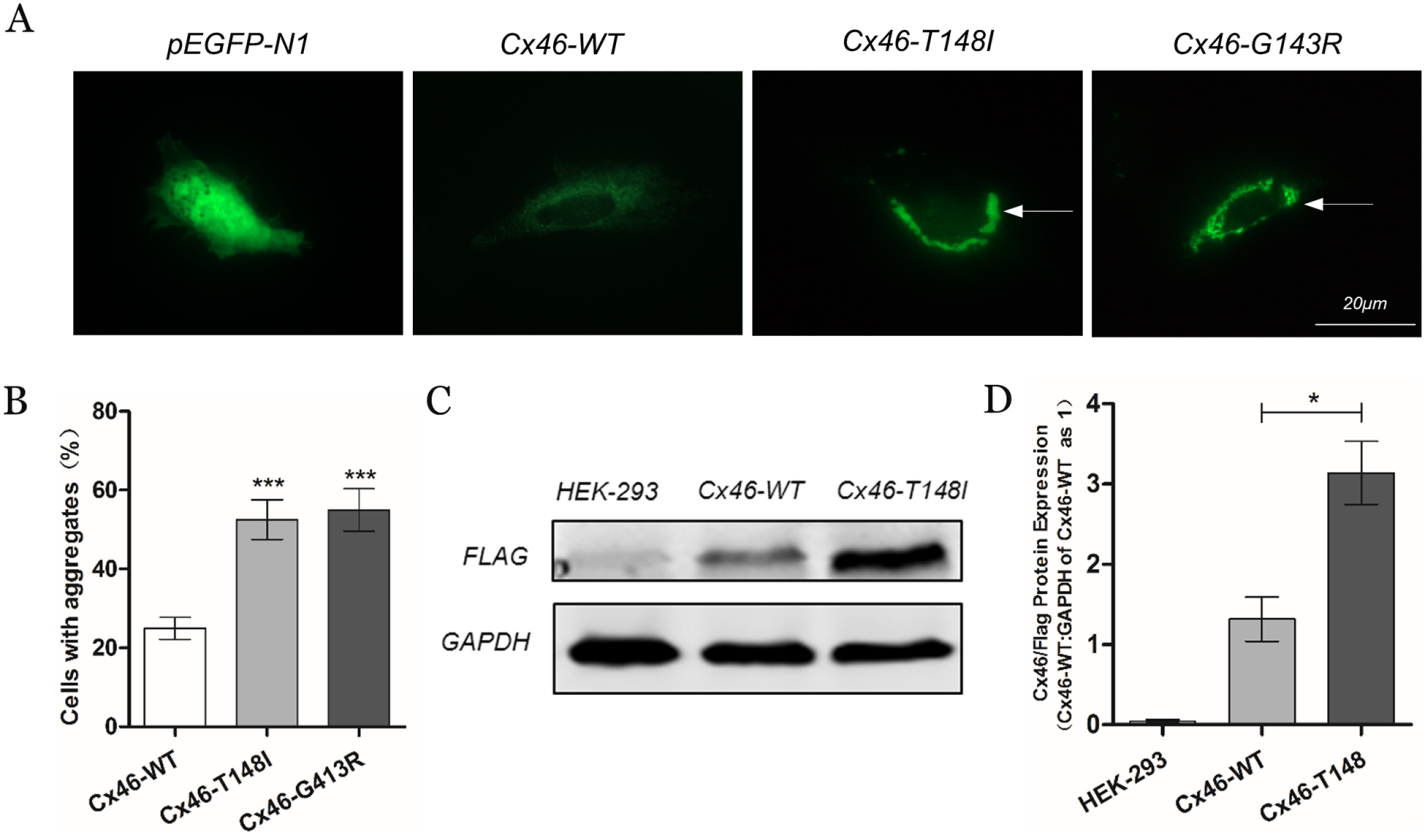
Fluorescence microscopy and Western blot analysis. (A) pEGFP-N1, Cx46WT, Cx46T148I and Cx46G143R EGFP-tagged proteins were located in the cytoplasm and plasma membrane of normal-density HLECs. However, Cx46T148I as well as the reported Cx46-G143R showed aggregate signals from cytoplasmic inclusions in fluorescent images rather than the typical punctate staining in the WT proteins. (B) Cx46T148I and Cx46-G143R significantly increased the intracellular aggregation. (C) and (D) Western blot analysis of the cell lysates indicated a higher expression level of mutant Cx46/Flag than the WT or control lanes. Scale bar:20 μm.

Inverted fluorescence microscope analysis showed that both EGFP-tag *GJA3* WT and T148I mutant proteins were located in the cytoplasm and plasma membrane of normal-density plated HLECs (Fig 6A). Intriguingly, both types of HELCs transfected with WT and T148I vectors could form GJ plaques when low-density cells were seeded (S1A Fig). Two kinds of plaques were located in the plasma membranes as linear distributions between cell-cell appositions. There were no significant differences between the WT and mutants when the percentages of plaque formation were compared between these three types (S1B Fig). However, mutant proteins showed aggregate signals in cytoplasmic inclusions rather than the typical punctate staining in the WT proteins (Fig 6A).

Western blot analysis indicated a higher expression level of mutant Cx46T148I than that of the WT or control lanes, which was in accordance with the accumulation of EGFP fluorescence signal intensity (Fig 6C and 6D). There is a reciprocal relationship between Cx43 and Cx46 expressed in the lens[30]. Meanwhile, HEK-293 cells and HLECs are also known to express Cx43. Our study shows that Cx43 has the same protein level among HEK-293, Cx46WT and Cx46T148I cells (S2 Fig). There was also no significant difference among transfected and non-transfected HLECs (S2 Fig). Therefore, the protein level of Cx43 may not be influenced by Cx46T148I in these cells. Furthermore, cell growth curves showed a higher rate of cell growth in stable lines expressing the mutant Cx46 protein, indicating that mutation T148I had a positive effect on cell growth (S3 Fig).

DAPI uptake assays showed that almost all the nuclei of HEK-293 (96.90±1.47%) and Cx46WT cells (96.57±0.87%) were labeled in blue by DAPI, whereas Cx46T148I cells (5.88±2.01%) contained only a small part of DAPI-stained cells after a 30 min incubation in Ca^2+^-free D-Hanks (Ca^2+^ free group in S4A Fig). A few HEK-293 (4.28±1.00%), Cx46WT (5.06±1.69%) and Cx46T148I cells (1.63±1.19%) were loaded with DAPI after incubation in D-Hanks containing 1.2 mM Ca^2+^ (1.2 mM Ca^2+^ group in S4A Fig). The same situation comes up in another group, a very small percentage of DAPI-stained HEK-293 (1.95±1.43%), Cx46WT cells (0.94±1.03%) and Cx46T148I cells (0.56±0.68%) were observed after treatment with D-Hanks containing 300 mM FFA (300 μM FFA group in S4A Fig).

## Discussion

In the present study, we enrolled a large ADCC family from southern China who were affected by bilateral pulverulent nuclear cataracts. A novel point mutation of *GJA3* in exon 2 at nucleotide 443 was identified via target exome capture sequencing, which led to a threonine-to-isoleucine change at amino acid 148 (p. T148I).

Thirty-two mutations identified in *GJA3* are associated with human inherited cataracts (summarized in S2 Table). Most of these mutations are missense mutations, except two insertions (c.1137insC and c.1361insC) and one deletion mutation (c.1143_1165del23). According to the anatomical locations of lens opacities, inherited cataracts can be categorized as capsular, cortical, embryonic, fetal, lamellar, nuclear, subcapsular, sutural or total cataract[7]. Given the genetic and clinical heterogeneity of inherited cataracts, establishing precise correlations between genotype and phenotype remains a challenge. Furthermore, the phenotypes of patients with *GJA3* mutations are diverse. Approximately half of the phenotypes are nuclear, another third are cortical, and the rest are capsular, lamellar, sutural or subcapsular. Interestingly, the majority of the mutations located in E1 and E2 domains of Cx46 are phenotypically nuclear cataracts, while phenotypes of CL domain mutations are nuclear or cortical. In this family, affected members had the similar pulverulent nuclear cataracts, which supports mutations in the CL domains causing the nuclear cataract phenotype. However, blurred vision gradually appeared after approximately age 10, which is earlier than other *GJA3* mutations. Their lens opacities appeared from the periphery to the center at subsequent stages. Therefore, operations had been implemented at age 10 or older in this family.

Our bioinformatic analysis and molecular consequences suggest that the *GJA3*/p. T148I mutation is linked to cataract formation in this ADCC family.

A high-quality TES was sufficient for the novel mutation screening and validation in this family based on the 1000 Genomes Project, ESP6500, ExAC, HGMD and the MyGenostics local database. The threonine at the 148^th^ amino acid (T148) of Cx46 was a highly conserved residue when the sequence was aligned with other vertebrates. Additionally, this mutation was co-segregated in affected members, while it was not detected in the 100 unaffected local Chinese controls. In a hydrophilic analysis, the substitution of a hydrophilic threonine by a hydrophobic isoleucine at position 148 might increase the hydrophobicity of Cx46, which is not conducive to the stable formation of Cx46 and underlines the importance of function in the cytoplasmic loop domain of this protein[31, 32]. Cx46 contains four transmembrane domains, including M1 to M4. Three loops, including two extracellular loops and one cytoplasmic loop, connect these four transmembrane domains, with an N-terminus and a C-terminus located on either side of the cytoplasmic domain[12]. It is worth noting that p. T148I was located in the cytoplasmic loop domain of the Cx46, but compared with the WT, homologous models were predicted to be an α-helix deletion in the C-terminus of the mutant Cx46 conformation (Fig 5B). The “ball-chain” balance that was important for the channel effect of Cx46 may be impaired by this α-helix deletion[33]. Hence, p. T148I can give rise to the abnormal Cx46 conformation and insolubility in the human lens.

Fluorescent images of EGFP-tagged *GJA3* WT and p. T148I fusion proteins showed that both Cx46 proteins were located in the cytoplasm and plasma membrane. However, there was no significant difference between the WT and mutant when the amounts were compared between these two types. Nevertheless, mutant proteins showed a high percentage of fusion-protein aggregation in the cytoplasm rather than the typical punctate staining in the WT proteins (Fig 6A and 6B), and aggregate signals in cells were similar to those of other *GJA3* or *GJA8* mutations[19, 34]. Moreover, protein expression in Cx46T148I was much higher than that in WT (Fig 6C and 6D), in accordance with the consequences of other connexin mutations[19, 35].

The results of the dye uptake experiment revealed that the opened hemichannels of Cx46T148I stable cells could not absorb DAPI, whereas HEK-293 and WT stable cells could (Ca^2+^ free group in S4A Fig). Meanwhile, both Cx46WT and Cx46T148I cells could form GJ plaques in the membranes (S1A Fig). Compared with Cx46WT cells, these results indicated that the hemichannel activity of Cx46T148I was strikingly decreased, although a similar amount of mutant plaque formation was observed[28].

Cx43 and Cx46 are the main connexins in human lens, and Cx43 are reported to be down-regulation by certain mutations of connexins [28, 36, 37]. In order to elucidate whether the Cx43 protein expression can be regulated by Cx46T148I, we detected the protein expression levels of Cx43 among HEK-293, Cx46WT and Cx46T148I cells. Fortunately, there was no significant difference among transfected and non-transfected HEK-293 cells, and similar expression levels were detected among HLECs (S2 Fig). It has been reported that Cx43 may not be crucial for normal function in lens, because the animals affected with a deletion of Cx43 were still showed transparent lens and normal development until mice were six months old [37, 38]. When expressed in cell lines, only a part of Cx43 and Cx46 are competent enough to form Cx43/Cx46 heteromeric complexes [39, 40]. In addition, the hemichannel function formed by heterotypic Cx43/Cx46 accounts for only a fraction of the total hemichannel functions. And hemichannels formed by Cx46 rather than heterotypic Cx43/Cx46 are responsible for the dye uptake in vitro[39, 41]. Hence, the hemichannel function of Cx46WT or T148I in present study might not be affected by Cx43.

Connexin may control cell growth, and certain mutant connexin protein exerts a dominant effect on cell proliferation[19, 42, 43]. Hence, a CCK-8 assay was performed to investigate the effects of Cx46T148I mutation on cell growth. Compared with Cx46WT cells, higher growth rates were observed in the cells of mutated Cx46T148I with stable ectopic expression (S3 Fig). These higher growth curves indicated that the Cx46T148I mutation has a positive effect on cell growth in vitro. We speculate that the higher rates of cell growth may partly explain the greater Cx46 expression derived from a positive effect on cells and that greater cell proliferation may also be caused by decreased hemichannel activity[35].

The Cx46T148I is a CL mutation and is predicted to cause an α-helix deletion in the C-terminus. The imbalance of the ball-chain interaction formed by mutated CL and C-terminus domains may contribute to this aberrant conformation, resulting in a reduction in GJ function[44]. Therefore, we presume that the loss of hemichannel function, along with the α-helix deletion of the C-terminus, may alter the metabolic balance of the lens and ultimately may cause cataract formation[43, 45].

In summary, a novel missense mutation c.443C>T in exon 2 of the *GJA3* gene was identified by TES. This discovery expands the spectrum of mutations resulting in ADCC. These data also suggest that TES is a time-saving and efficient method for the molecular diagnosis of ADCC, which shows clinical and genetic heterogeneity. Additionally, this novel mutation caused an α-helix deletion in the C-terminus of Cx46 as well as abnormal protein aggregation retained in the cytoplasm, and the increased level of mutant protein may be caused by this change in molecular conformation. Meanwhile, hemichannel activity was also influenced by Cx46T148I. These results can be interpreted as part of the molecular mechanism that causes cataract formation in this ADCC family. However, since Cx46 has multiple functions, more evidence is needed to elucidate the pathophysiologic changes in GJs caused by this mutation.

## Supporting information

S1 Fig Gap junction plaques in low-density cells. (A) Plaques were located in the plasma membranes as the linear distribution between cell-cell appositions (white arrows). (B) There was no significant difference between WT and mutant when the amounts were compared between both types. Scale bar:20 μm.
(TIF)

S2 Fig Endogenous expression of Cx43 in HEK-293 cells and HLECs. (A) Cx43 protein are similarly expressed among HEK-293, Cx46WT and Cx46T148I cells. (B) and (C) There are no significant differences among transfected and non-transfected HLECs. Therefore, Cx43 protein may not be influenced by Cx46T148I in these cells.
(TIF)

S3 Fig Cell growth curve of the WT and mutated *GJA3*. *GJA3*/p. T148I has a positive effect on HEK-293 cell growth.
(TIF)

S4 Fig DAPI dye uptake in HEK-293 cells stably transfected with Cx46WT and Cx46T148I. Cells were immunostained with anti-Flag monoclonal antibody after DAPI dye uptake procedure. Cx46/Flag positive cells (Cx46-WT and Cx46-T148I groups) were showed in green fluorescence while HEK-293 group showed none. (A) Ca^2+^ free group: DAPI was absorbed by most of the HEK-293 and Cx46WT cells but only a small part by the Cx46T148I cells after a 30 min incubation in Ca^2+^-free D-Hanks solution; 1.2 mM Ca^2+^ group and 300 μM FFA group: DAPI loading in Ca^2+^-free HBSS was blocked by 1.2 mM Ca^2+^ and 300 mM FFA. (B) Statistical analysis of DAPI-stained cells in different incubation solutions. Data are presented as the mean±SDs. There was a significant difference in the percentage of dye-stained cells between the Cx46WT and Cx46T148I groups in the Ca^2+^-free medium (*P*<0.001). Almost all the nuclei of HEK-293 (96.90±1.47%) and Cx46WT cells (96.57±0.87%) were labeled in blue by DAPI, whereas Cx46T148I cells (5.88±2.01%) contained only a few DAPI-stained cells after incubation in Ca^2+^-free D-Hanks solution (Ca^2+^ free group). Few HEK-293 (4.28±1.00%), Cx46WT cells (5.06±1.69%) and Cx46T148I cells (1.63±1.19%) were loaded with DAPI after incubation in D-Hanks containing 1.2 mM Ca^2+^ (1.2 mM Ca^2+^ group). The same result was observed in another group. Few DAPI-stained HEK-293 (1.95±1.43%), Cx46WT (0.94±1.03%) and Cx46T148I cells (0.56±0.68%) were observed after treatment with D-Hanks containing 300 mM FFA (300 μM FFA group). Scale bar:50 μm.
(TIF)

S1 Table A total of 134 genes captured in this study. (PDF)

S2 Table Mutations in *GJA3* associated with human CC. (PDF)
