# Supplementary figures and images for: Identification of a novel GJA3 mutation in a large Chinese family with congenital cataract using targeted exome sequencing
Source: PLoS One. 2017 Sep 6;12(9):e0184440. doi: 10.1371/journal.pone.0184440 (PMC5587237; doi:10.1371/journal.pone.0184440)

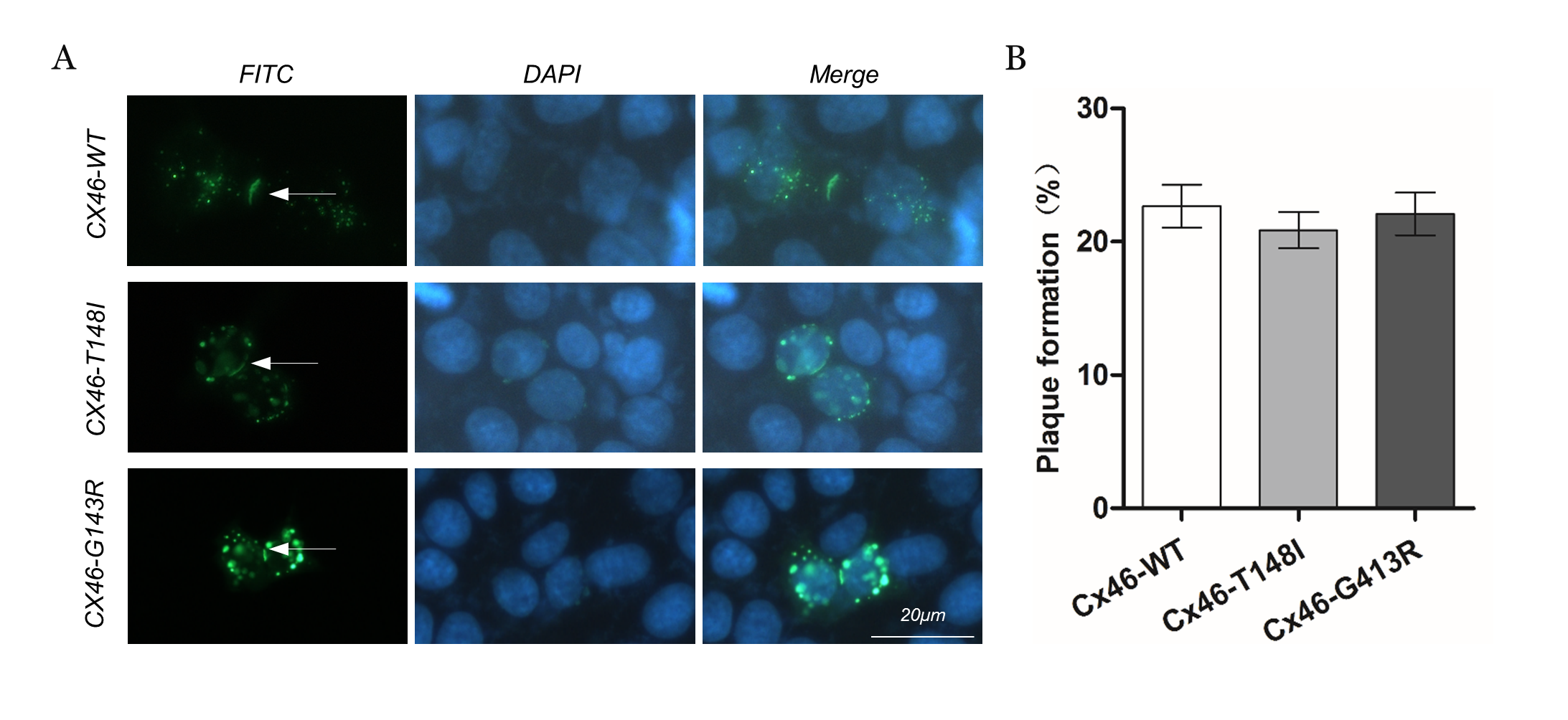

Supplement: S1 Fig — (A) Plaques were located in the plasma membranes as the linear distribution between cell-cell appositions (white arrows). (B) There was no significant difference between WT and mutant when the amounts were compared between both types. Scale bar:20 μm. (TIF) [file pone.0184440.s001.tif]

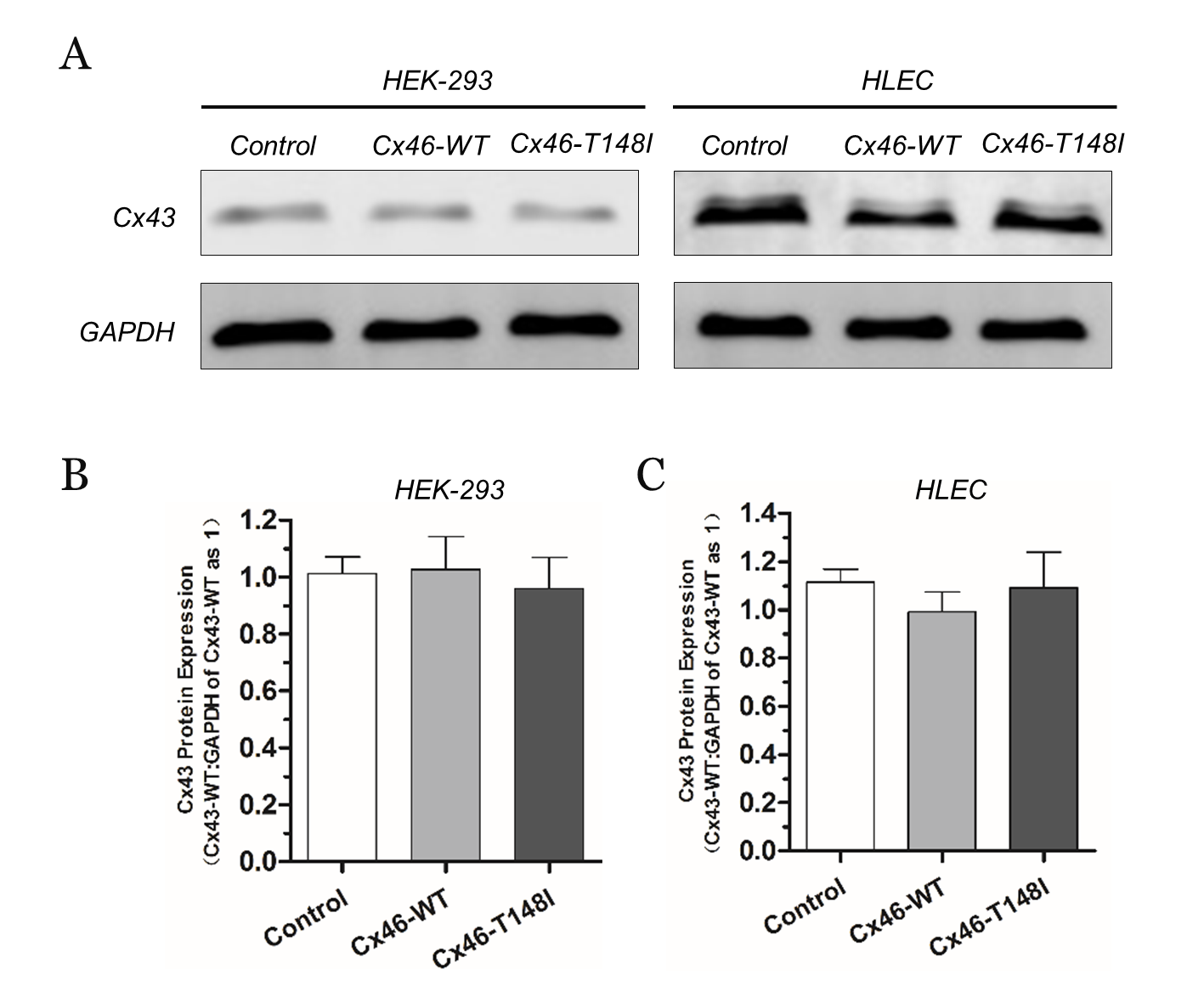

Supplement: S2 Fig — (A) Cx43 protein are similarly expressed among HEK-293, Cx46WT and Cx46T148I cells. (B) and (C) There are no significant differences among transfected and non-transfected HLECs. Therefore, Cx43 protein may not be influenced by Cx46T148I in these cells. (TIF) [file pone.0184440.s002.tif]

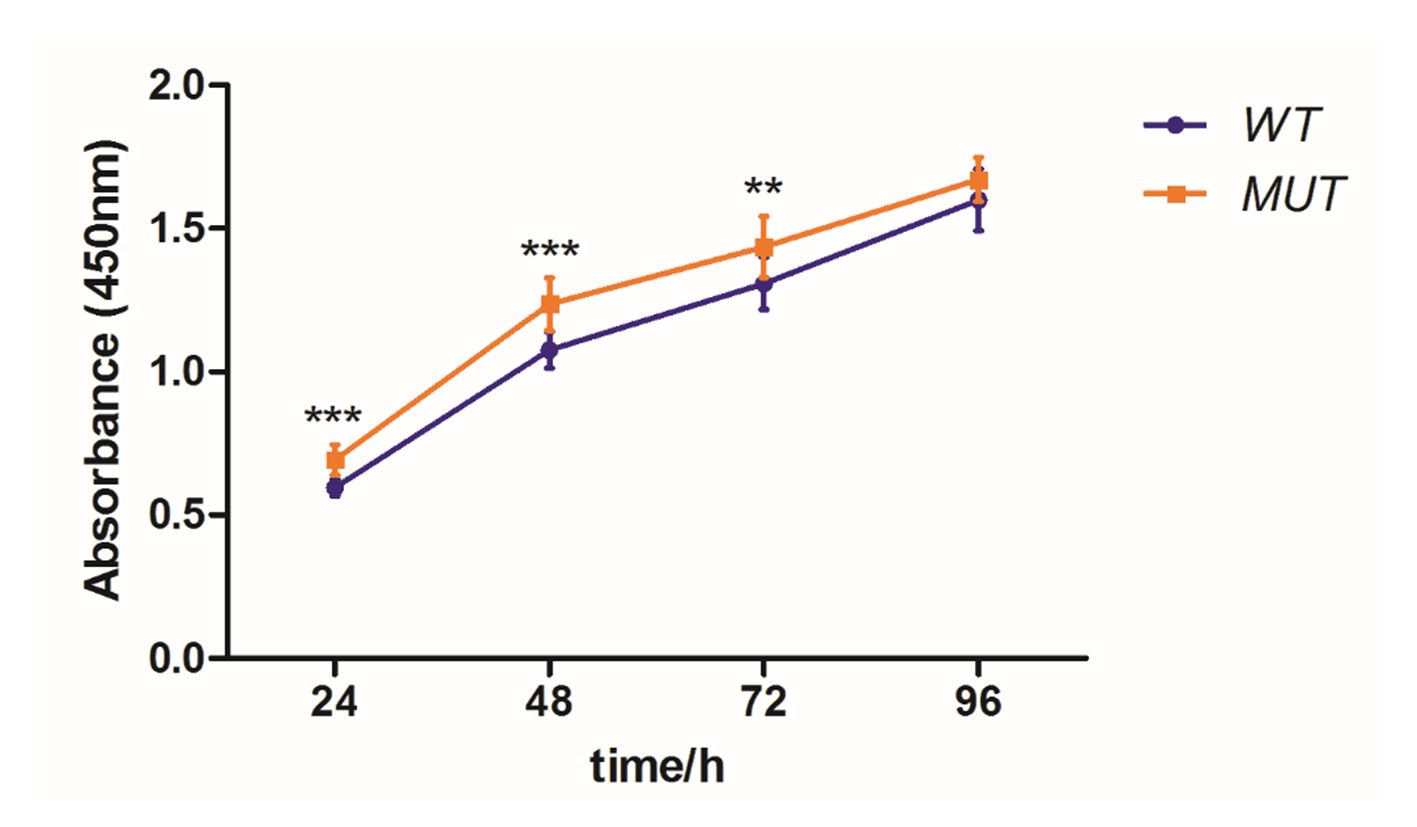

Supplement: S3 Fig — GJA3/p. T148I has a positive effect on HEK-293 cell growth. (TIF) [file pone.0184440.s003.tif]

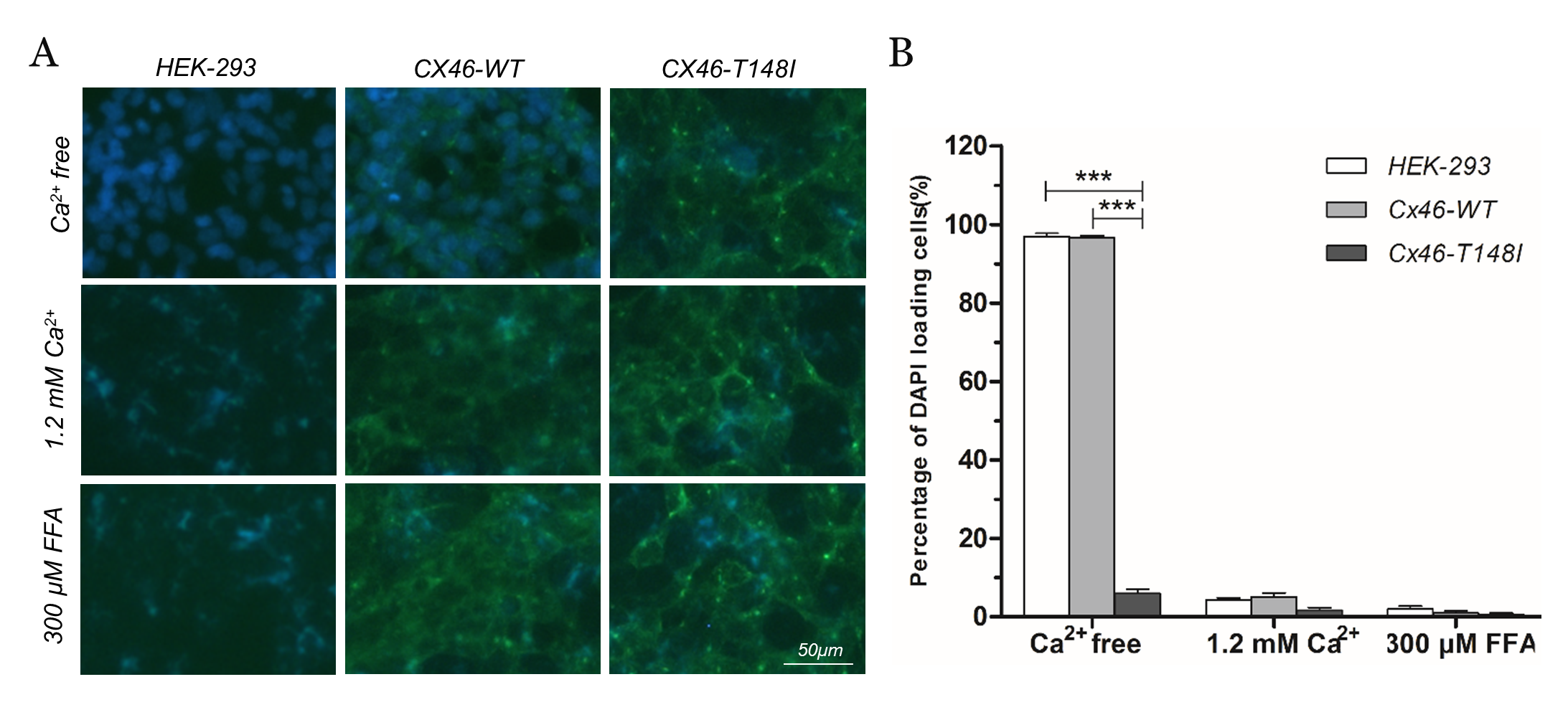

Supplement: S4 Fig — Cells were immunostained with anti-Flag monoclonal antibody after DAPI dye uptake procedure. Cx46/Flag positive cells (Cx46-WT and Cx46-T148I groups) were showed in green fluorescence while HEK-293 group showed none. (A) Ca2+ free group: DAPI was absorbed by most of the HEK-293 and Cx46WT cells but only a small part by the Cx46T148I cells after a 30 min incubation in Ca2+-free D-Hanks solution; 1.2 mM Ca2+ group and 300 μM FFA group: DAPI loading in Ca2+-free HBSS was blocked by 1.2 mM Ca2+ and 300 mM FFA. (B) Statistical analysis of DAPI-stained cells in different incubation solutions. Data are presented as the mean±SDs. There was a significant difference in the percentage of dye-stained cells between the Cx46WT and Cx46T148I groups in the Ca2+-free medium (P<0.001). Almost all the nuclei of HEK-293 (96.90±1.47%) and Cx46WT cells (96.57±0.87%) were labeled in blue by DAPI, whereas Cx46T148I cells (5.88±2.01%) contained only a few DAPI-stained cells after incubation in Ca2+-free D-Hanks solution (Ca2+ free group). Few HEK-293 (4.28±1.00%), Cx46WT cells (5.06±1.69%) and Cx46T148I cells (1.63±1.19%) were loaded with DAPI after incubation in D-Hanks containing 1.2 mM Ca2+ (1.2 mM Ca2+ group). The same result was observed in another group. Few DAPI-stained HEK-293 (1.95±1.43%), Cx46WT (0.94±1.03%) and Cx46T148I cells (0.56±0.68%) were observed after treatment with D-Hanks containing 300 mM FFA (300 μM FFA group). Scale bar:50 μm. (TIF) [file pone.0184440.s004.tif]
