# Supplementary material for: Identification of a novel GJA3 mutation in a large Chinese family with congenital cataract using targeted exome sequencing
Source: PLoS One. 2017 Sep 6;12(9):e0184440. doi: 10.1371/journal.pone.0184440 (PMC5587237; doi:10.1371/journal.pone.0184440)
Supplement: S1 Table — (PDF) [file pone.0184440.s005.pdf]

Supplementary Table 1. 134 genes captured in this study.

ABHD12	*CRYAA*	*ESR1*	*GSTT1*	*NDP*	*PEX7*	*SOX2*
ADAM9	*CRYAB*	*EYA1*	*HMX1*	*NF2*	*PITX2*	*SRD5A3*
ADAMTS10	*CRYBA1*	*EZR*	*HSF4*	*NHS*	*PITX3*	*SREBF2*
ADAMTSL4	*CRYBA2*	*FAM126A*	*IDO1*	*NOG*	*PVRL3*	*TDRD7*
AGK	*CRYBA4*	*FBN1*	*IFNGR1*	*OAT*	*PXDN*	*TFAP2A*
AGPS	*CRYBB1*	*FOXC1*	*ITM2B*	*OCRL*	*RAB18*	*TGFB3*
ALDH18A1	*CRYBB2*	*FOXD3*	*JAM3*	*OPA3*	*RAB3GAP1*	*TMEM114*
APOE	*CRYBB3*	*FOXE3*	*KCNJ13*	*PAX6*	*RAB3GAP2*	*TMEM70*
ATOH7	*CRYGB*	*FTL*	*LCA5*	*PEX10*	*RECQL4*	*UNC45B*
B3GALTL	*CRYGC*	*FTO*	*LCT*	*PEX11B*	*RNLS*	*VIM*
BCOR	*CRYGD*	*FYCO1*	*LIM2*	*PEX12*	*SC5DL*	*VLDLR*
BEST1	*CRYGS*	*FZD4*	*LMX1B*	*PEX13*	*SEC23A*	*VSX2*
BFSP1	*CTDP1*	*GALK1*	*LRP5*	*PEX14*	*SIL1*	*WFS1*
BFSP2	*CTNND2*	*GALT*	*MAF*	*PEX16*	*SIX5*	*WRN*
CHMP4B	*CYP27A1*	*GFER*	*MAN2B1*	*PEX19*	*SIX6*	
COL11A1	*CYP51A1*	*GJA1*	*MIP*	*PEX2*	*SLC16A12*	
COL18A1	*DHCR7*	*GJA3*	*MTHFR*	*PEX26*	*SLC25A15*	
COL2A1	*EPG5*	*GJA8*	*MVK*	*PEX3*	*SLC2A1*	
COL4A1	*EPHA2*	*GNPAT*	*MYH9*	*PEX5L*	*SLC33A1*	
COL7A1	*ERCC2*	*GSTM1*	*NAT2*	*PEX6*	*SLC7A14*	
