# Supplementary material for: Identification of a novel GJA3 mutation in a large Chinese family with congenital cataract using targeted exome sequencing
Source: PLoS One. 2017 Sep 6;12(9):e0184440. doi: 10.1371/journal.pone.0184440 (PMC5587237; doi:10.1371/journal.pone.0184440)
Supplement: S2 Table — (PDF) [file pone.0184440.s006.pdf]

Supplementary Table 2. Mutations in *GJA3* associated with human CC.

No.	Nucletide change	AA change	Domain	Cataract phenotype	Mechanisms	Reference
1	c.1A>G	p.M1V	N	Anterior polar	Predict: formation of a truncated protein	[1]
2	c.5G>A	p.G2D	N	Nuclear pulverulent and posterior polar	Gap junction plaques inefficiently;<br>Changed hemichannel permeability;<br>Caused apoptosis	[2]
3	c.7G>T	p.D3Y	N	Zonular pulverulent	has not been studied yet	[3]
4	c.32T>C	p.L11S	N	"Ant-egg"	has not been studied yet	[4]
5	c.56C>T	p.T19M	N	Posterior-polar	Loss of gap junction function;<br>Alters hemi-channel gating	[5, 6]
6	c.82G>A	p.V28M	M1	Total	has not been studied yet	[7]
7	c.96C>A	p.F32L	M1	Nuclear	has not been studied yet	[8]
8	c.98G>T	p.R33L	M1	Finely granular embryonal	has not been studied yet	[9]
9	c.125A>C	p.E42A	E1	Pulverulent	has not been studied yet	[10]
10	c.130G>A	p.V44M	E1	Nuclear	has not been studied yet	[11, 12]
11	c.134G>C	p.W45S	E1	Nuclear	has not been studied yet	[13]
12	c.139G>A	p.D47N	E1	Nuclear	has not been studied yet	[14]
13	c.143A>G	p.E48G	E1	Nuclear	Predict: formation of a truncated protein	[15]
14	c.163A>G	p.N55D	E1	Nuclear	Affect formation of hydrogen bonds;<br>Lead to an unstable conformation.	[16]
15	c.176C>T	p.P59L	E1	Nuclear punctate	has not been studied yet	[17-19]
16	c.188A>G	p.N63S	E1	Zonular pulverulent	has not been studied yet	[20]
17	c.226C>G	p.R76G	E1	Anterior capsular/posterior cortical opacities	has not been studied yet	[7]
18	c.227G>A	p.R76H	E1	Nuclear pulverulent/lamellar and suture	has not been studied yet	[21, 22]
19	c.260C>T	p.T87M	M2	Pearl box	has not been studied yet	[9]
20	c.268C>T	p.L90F	M2	Pulverulent	has not been studied yet	[10]
21	c.415G>A	p.V139M	CL	Cortical	has not been studied yet	[23]
22	c.427G>A	p.G143R	CL	Coppock-like	Alters its interaction with C-terminal domain and calmodulin;<br>Gating of hemichannels	[24]
23	c.428G>A	p.G143E	CL	Nuclear	has not been studied yet	[25]
24	c.559C>T	p.P187S	E2	Nuclear pulverulent	has not been studied yet	[26]
25	c.560C>T	p.P187L	E2	Zonular pulverulent	has not been studied yet	[27]
26	c.563A>C	p.N188T	E2	Nuclear pulverulent	has not been studied yet	[28]
27	c.563A>T	p.N188I	E2	Nuclear coralliform/zonular pulverulent	Predict: affect β sheet at amino acid	[29]
28	c.589C>T	p.P197S	E2	Nuclear	has not been studied yet	[30]
29	c.616T>A	p.F206I	M4	Nuclear	has not been studied yet	[31]
30	c.1137insC	-	C	Zonular pulverulent	has not been studied yet	[20]
31	c.1143_1165del23	-	C	Punctate nuclear	has not been studied yet	[32]
32	c.1361insC	-	C	Coralliform	has not been studied yet	[33]

References

1. Kaur P, Kumar M, Khokhar S, Dada R.Molecular and structural analysis of genetic variations in congenital cataract[J].Mol Vis.2013,19(2):2436-2450.
2. Yao K, Wang W, Zhu Y, Jin C, Shentu X, Jiang J, Zhang Y, Ni S.A novel GJA3 mutation associated with congenital nuclear pulverulent and posterior polar cataract in a Chinese family[J].Hum Mutat.2011,32(12):1367–1370.
3. Addison PK, Berry V, Holden KR, Espinal D, Rivera B, Su H, Srivastava AK, Bhattacharya SS.A novel mutation in the connexin 46 gene (GJA3) causes autosomal dominant zonular pulverulent cataract in a Hispanic family[J].Mol Vis.2006,12(88-90):791.
4. Hansen L, Yao W, Eiberg H, Funding M, Riise R, Kjaer KW, Hejtmancik JF, Rosenberg T. The congenital "ant-egg" cataract phenotype is caused by a missense mutation in connexin46[J].Mol Vis.2006,12(12):1033-1039.
5. Santhiya ST, Kumar GS, Sudhakar P, Gupta N, Klopp N, Illig T, Söker T, Groth M, Platzer M, Gopinath PM. Molecular analysis of cataract families in India: new mutations in the CRYBB2 and GJA3 genes and rare polymorphisms[J]. Mol Vis.2010,16(199):1837-1847.
6. Tong JJ, Minogue PJ, Kobeszko M, Beyer EC, Berthoud VM, Ebihara L. The Connexin46 Mutant, Cx46T19M, Causes Loss of Gap Junction Function and Alters Hemi-channel Gating[J].The Journal of Membrane Biology.2015,248(1):145.
7. Devi RR, Reena C, Vijayalakshmi P. Novel mutations in GJA3 associated with autosomal dominant congenital cataract in the Indian population[J]. Mol Vis.2005,11(100-1):846-852.
8. Jiang H, Jin Y, Bu L, Zhang W, Liu J, Cui B, Kong X, Hu L. A novel mutation in GJA3 (connexin46) for autosomal dominant congenital nuclear pulverulent cataract[J]. Mol Vis.2003,9(70):579-583.
9. Guleria K, Sperling K, Singh D, Varon R, Singh JR, Vanita V. A novel mutation in the connexin 46 (GJA3) gene associated with autosomal dominant congenital cataract in an Indian family[J].Mol Vis.2007,13(183-85):1657-1665.
10. Yang Z, Li Q, Ma X, Zhu SQ. Mutation Analysis in Chinese Families with Autosomal Dominant Hereditary Cataracts[J]. Curr Eye Res.2015,40(12):1.
11. Bennett TM, Shiels A. A recurrent missense mutation in GJA3 associated with autosomal

dominant cataract linked to chromosome 13q[J]. Mol Vis.2011,17(1):2255.

12. Zhou Z, Hu S, Wang B, Zhou N, Zhou S, Xu M, Qi Y. Mutation analysis of congenital cataract in a Chinese family identified a novel missense mutation in the connexin 46 gene (GJA3) [J].Mol Vis.2010,16:713-719.
13. Ma ZW, Zheng JQ, Li J, Li XR, Tang X, Yuan XY, Zhang XM, Sun HM. Two novel mutations of connexin genes in Chinese families with autosomal dominant congenital nuclear cataract[J]. Br J Ophthalmol.2005,89(11):1535-1537.
14. Guo Y, Yuan L, Yi J, Xiao J, Xu H, Lv H, Xiong W, Zheng W, Guan L, Zhang J. Identification of a GJA3 mutation in a Chinese family with congenital nuclear cataract using exome sequencing[J]. Indian J Biochem Biophys.2013,50(4):253.
15. Li B, Liu Y, Liu Y, Guo H, Hu Z, Xia K, Jin X. Identification of a GJA3 Mutation in a Large Family with Bilateral Congenital Cataract[J]. DNA Cell Biol.2015,35(3).
16. Hu Y, Gao L, Feng Y, Yang T, Huang S, Shao Z, Yuan H. Identification of a novel mutation of the gene for gap junction protein α3 (GJA3) in a Chinese family with congenital cataract[J].Mol Biol Rep.2014,41(7):4753-4758.
17. Hansen L, Mikkelsen A, Nürnberg P, Nürnberg G, Anjum I, Eiberg H, Rosenberg T. Comprehensive mutational screening in a cohort of Danish families with hereditary congenital cataract[J]. IOVS.2009,50(7):3291.
18. Bennett TM, Mackay DS, Knopf HL, Shiels A. A novel missense mutation in the gene for gap-junction protein alpha3 (GJA3) associated with autosomal dominant "nuclear punctate" cataracts linked to chromosome 13q[J]. Mol Vis.2004,10(1):376.
19. Wenmin Sun XX, Shiqiang Li, Xiangming Guo, Qingjiong Zhang.Mutation analysis of 12 genes in Chinese families with congenital cataracts[J]. Mol Vis.2011,17(238-39):2197-2206.
20. Mackay D, Ionides A, Kibar Z, Rouleau G, Berry V, Moore A, Shiels A, Bhattacharya S. Connexin46 mutations in autosomal dominant congenital cataract[J]. Am J Hum Genet.1999,64(5):1357.
21. Hansen L, Mikkelsen A, Nürnberg P, Nürnberg G, Anjum I, Eiberg H, Rosenberg T. Comprehensive mutational screening in a cohort of Danish families with hereditary congenital cataract[J].IOVS.2009,50(7):3291-3303.
22. Burdon KP, Wirth MG, Mackey DA, Russelleggitt IM, Craig JE, Elder JE, Dickinson JL, Sale

MM. Investigation of crystallin genes in familial cataract, and report of two disease associated mutations[J].Br J Ophthalmol.2004,88(1):79-83.

23. Zhou Z, Wang B, Hu S, Zhang C, Xu M, Qi Y. Genetic variations in GJA3, GJA8, LIM2, and age-related cataract in the Chinese population: a mutation screening study[J]. Mol Vis.2011,17(70-71):621-626.
24. Zhang L, Qu X, Su S, Guan L, Liu P.A novel mutation in GJA3 associated with congenital Coppock-like cataract in a large Chinese family[J]. Mol Vis.2012,18(220-25):2114-2118.
25. Yuan L, Guo Y, Yi J, Xiao J, Yuan J, Xiong W, Xu H, Yang Z, Zhang J, Deng H. Identification of a novel GJA3 mutation in congenital nuclear cataract[J]. Optometry & Vision Science Official Publication of the American Academy of Optometry.2015,92(3):337.
26. Ding X, Wang B, Luo Y, Hu S, Zhou G, Zhou Z, Jing W, Xu M, Qi Y. A novel mutation in the connexin 46 (GJA3) gene associated with congenital cataract in a Chinese pedigree[J]. Mol Vis.2011,17(151):1343.
27. Rees MI, Watts P, Fenton I, Clarke A, Snell RG, Owen MJ, Gray J. Further evidence of autosomal dominant congenital zonular pulverulent cataracts linked to 13q11 (CZP3) and a novel mutation in connexin 46 (GJA3) [J]. Hum Genet.2000,106(2):206-209.
28. Li Y, Wang J, Dong B, Man H. A novel connexin46 (GJA3) mutation in autosomal dominant congenital nuclear pulverulent cataract[J]. Mol Vis.2004,10:668.
29. Zhang X, Wang L, Wang J, Dong B, Li Y. Coralliform cataract caused by a novel connexin46 (GJA3) mutation in a Chinese family[J]. Mol Vis.2012,18(18):203-210.
30. Ponnam SP, Ramesha K, Matalia J, Tejwani S, Ramamurthy B, Kannabiran C. Mutational screening of Indian families with hereditary congenital cataract[J]. Mol Vis.2013,19(2):1141-1148.
31. Wang KJ, Zhu SQ. A novel p. F206I mutation in Cx46 associated with autosomal dominant congenital cataract[J].Mol Vis.2012,18(99-102):968.
32. Sun W, Xiao X, Li S, Guo X, Zhang Q.Mutation analysis of 12 genes in Chinese families with congenital cataracts[J].Mol Vis.2011,17(238-39):2197.
33. Zhou D, Ji H, Wei Z, Guo L, Li Y, Wang T, Zhu Y, Dong X, Wang Y, Lin H.A novel insertional mutation in the connexin 46 (gap junction alpha 3) gene associated with autosomal dominant congenital cataract in a Chinese family[J].Mol Vis.2013,19(7):789-795.
